# GNL3L Is a Nucleo-Cytoplasmic Shuttling Protein: Role in Cell Cycle Regulation

**DOI:** 10.1371/journal.pone.0135845

**Published:** 2015-08-14

**Authors:** Indu Jose Thoompumkal, Malireddi Rama Krishna Subba Rao, Anbarasu Kumaraswamy, Rehna Krishnan, Sundarasamy Mahalingam

**Affiliations:** 1 Laboratory of Molecular Virology and Cell Biology, Department of Biotechnology, Bhupat and Jyoti Mehta School of Biosciences, Indian Institute of Technology-Madras, Chennai, 600 036, India; 2 National Cancer Tissue Biobank, Department of Biotechnology, Bhupat and Jyoti Mehta School of Biosciences, Indian Institute of Technology-Madras, Chennai, 600 036, India; University of Toronto, CANADA

## Abstract

GNL3L is an evolutionarily conserved high molecular weight GTP binding nucleolar protein belonging to HSR1-MMR1 subfamily of GTPases. The present investigation reveals that GNL3L is a nucleo-cytoplasmic shuttling protein and its export from the nucleus is sensitive to Leptomycin B. Deletion mutagenesis reveals that the C-terminal domain (amino acids 501–582) is necessary and sufficient for the export of GNL3L from the nucleus and the exchange of hydrophobic residues (M567, L570 and 572) within the C-terminal domain impairs this process. Results from the protein-protein interaction analysis indicate that GNL3L interaction with CRM1 is critical for its export from the nucleus. Ectopic expression of GNL3L leads to lesser accumulation of cells in the ‘G2/M’ phase of cell cycle whereas depletion of endogenous GNL3L results in ‘G2/M’ arrest. Interestingly, cell cycle analysis followed by BrdU labeling assay indicates that significantly increased DNA synthesis occurs in cells expressing nuclear export defective mutant (GNL3L^∆NES^) compared to the wild type or nuclear import defective GNL3L. Furthermore, increased hyperphosphorylation of Rb at Serine 780 and the upregulation of E2F1, cyclins A2 and E1 upon ectopic expression of GNL3L^∆NES^ results in faster ‘S’ phase progression. Collectively, the present study provides evidence that GNL3L is exported from the nucleus in CRM1 dependent manner and the nuclear localization of GNL3L is important to promote ‘S’ phase progression during cell proliferation.

## Introduction

G-proteins (Guanine nucleotide binding proteins) function as molecular switches controlling several key cellular events owing to their inherent capacity to hydrolyze nucleotide triphosphates [1, 2]. Guanine nucleotide binding protein-like 3-like (GNL3L), characterized by nucleolar distribution, is a putative nucleolar GTPase belonging to the YawG/Y1qF/HSR1_MMR1 GTP-binding protein subfamily of GTPases. The proteins belonging to this group are characterized by a circular permutation of their GTP binding signature motifs (G1-G5) such that the G4 and G5 sub-domains are relocated from the C-terminus to the N-terminus of the protein [3, 4]. GNL3L encodes a polypeptide of 582 amino acids with a predicted molecular mass of 65 kDa. Grn1, the yeast homologue of GNL3L is required for growth and proliferation of *S*. *pombe* and the growth defect of Grn1-null mutant could be rescued by human GNL3L [5]. Reports suggest that GNL3L could have a tumor promoting role by binding and stabilizing MDM2 [6]. GNL3L inhibits Estrogen-related receptor gamma (ERR-gamma) activity by blocking the activity of steroid receptor co-activator (SRC) [7]. Telomere repeat binding factor (TRF1) was also found to interact with GNL3L and modulate metaphase to anaphase progression [8]. GNL3L interacts with importin-beta through its lysine-rich Nucleolar Localization Signal (NoLS) in the N-terminal region, which is distinct from other known NoLSs and is capable of transporting heterologous proteins to the nucleolus [9]. Interestingly, a functional NLS has also been identified between amino acids 51–100 in the N-terminal region, which interacts specifically with importin-alpha [9]. Recent report from our laboratory suggests that GNL3L exhibits predominant nucleolar localization in interphase cells (with relatively weak nuclear distribution) and this pattern was altered upon treatment with MPA (a GTP synthesis inhibitor) or Actinomycin-D (transcriptional inhibitor) [9]. This altered distribution of GNL3L from nucleolus to nuclear and cytoplasmic compartments raises the possibility that GNL3L shuttles between these compartments and the intracellular GTP pool may play a critical role in this process. The dynamics of nucleolar-nucleoplasmic shuttling of GNL3L has been described in detail elsewhere [10] but the mechanism and functional importance of its nucleo-cytoplasmic transport with respect to cell proliferation remains unknown.

Differential subcellular localization of the proteins is associated with diverse outcomes and delineation of nucleo-cytoplasmic transport of such proteins sheds light on their plausible biological functions. Transport of proteins, RNA and ribosomal subunits across the nuclear pore complex (NPC) is a receptor mediated process that occurs via the formation of RanGTP/RanGDP gradient, which is energy dependent. The karyopherin-β family of receptors which includes importins and exportins mediate most of the nucleo-cytoplasmic pathways within the cell. The shuttling between nucleus and cytoplasm has been demonstrated for nucleolar proteins such as nucleolin and nucleophosmin [11]. Such a process could serve as a regulatory mechanism for their nuclear functions or have a role in the nucleo-cytoplasmic transport of ribosomal subunits. Contrary to the earlier observations stating that specific domains were not required for nuclear export [12], nuclear export signals (NES) were found in diverse types of proteins as NMD3 [13,14] and cyclin B1 [15]. The most widely studied NES is a leucine-rich sequence initially discovered in HIV-1 Rev protein [16] and later in the cellular protein kinase inhibitor PKI [17]. Leptomycin-B (LMB), an antifungal metabolite isolated from *Streptomyces* efficiently inhibits nuclear export of HIV-1 Rev protein and Rev-dependent mRNA [18] and has been used extensively to study the nuclear export process. Chromosome Region Maintenance 1 (CRM1) is the cellular target for LMB, thus defined as the export receptor for leucine-rich NESs [19, 20, 21].

Given the increasing importance of nucleolar proteins in the regulation of cell growth and proliferation, we have attempted to address the relationship between nucleo-cytoplasmic transport of GNL3L and its functional significance during cell proliferation. The present study demonstrates that GNL3L is exported from the nucleus by a CRM1 dependent pathway. The C-terminal domain of GNL3L carries the leucine-rich signal required for its export from the nucleus. The abrogation of GNL3L export from the nucleus leads to increase in Rb phosphorylation on S780 and subsequent upregulation of E2F1 and its target genes, cyclins A2 and E1, resulting in faster ‘S’ phase progression during cell proliferation.

## Materials and Methods

### Plasmid Construction

GNL3L open reading frame was amplified from a HeLa cDNA library using appropriate primers (S1 Table) and cloned into pcDNA3 vector as a C- terminal enhanced green fluorescent protein (eGFP) fusion in EcoRI and EcoRV sites as described previously [9]. GNL3L was also cloned into pcDNA3 as N-terminal fusion with Flag-tag in KpnI and XhoI sites. The plasmids encoding GNL3L variants (residues 101–582, 201–582, 301–582, 401–582, 501–582, 51–500, 51–547) were generated using full-length GNL3L as template and sub-cloned into pcDNA3 as C-terminal eGFP fusion [9]. Site-specific mutants GNL3L^∆NES^ [GNL3L^1–582(M567,L570,572A)^, GNL3L^51–582(M567,L570,572A)^, GNL3L^501–582(M567,L570,572A)^] and GNL3L^∆NLS^ [GNL3L^1–582(K81,R82,83A)^] were generated by Quick-change mutagenesis (Stratagene, USA) according to manufacturer’s instructions using Flag-GNL3L^1–582^ or GNL3L^51–582^-GFP or GNL3L^501–582^-GFP as templates using appropriate primers (S1 Table). HA-tagged Rb expression plasmid (#10720–413) was obtained from Addgene, USA. The integrity of all the constructs was confirmed by DNA sequencing.

### Cell Culture, Transfection, Immunoprecipitation and Western blot analysis

HEK293[22], HeLa[23], COS-7[24], HepG2[25], Hep3B[25], MCF-7[26], and NIH-3T3[27] cells were maintained in Dulbecco’s Modified Eagle’s Medium (DMEM) (Invitrogen Life Technologies, USA) supplemented with 10% Fetal Bovine Serum (FBS) and 1% antibiotic-antimycotic (Invitrogen Life Technologies, USA). HeLa, COS-7 or HepG2 cells were infected with vTF7-3 [28], a recombinant vaccinia virus encoding bacteriophage T7 RNA polymerase, before being transfected with wild type or variants of GNL3L using lipofectin (Invitrogen Life Technologies, USA). HEK293 cells were grown to 60% confluency on 60-mm diameter dishes and transfection was carried out using lipofectamine (Invitrogen Life Technologies, USA) according to manufacturer’s protocol. MCF-7 cells were grown to 80% confluency and transfection was achieved by Lipofectamine 2000 (Invitrogen Life Technologies, USA). Hep3B cells were transfected using Xtremegene HD (Roche, USA) at 60–70% confluency according to manufacturer’s protocol. Transient knockdown of GNL3L was performed by lipofectamine 2000-mediated transfection of siRNA in HEK293 cells (L-015743-01-0005, ON-TARGET plus SMARTpool, Human GNL3L (54552), Thermo Fisher Scientific Inc., USA). The universal negative control siRNA (MISSION siRNA Universal Negative Control #1, SIC001, Sigma-Aldrich, USA) was used as a control for the knockdown studies.

For western blotting, transfected cells were solubilized in 1X cell lysis buffer (25 mM Tris-HCl, pH7.4, 150 mM KCl, 1 mM Na_2_EDTA, 1 mM EGTA, 1% Triton-X100, 2.5 mM sodium pyrophosphate, 1 mM *β*-glycerophosphate, 0.4 mM PMSF, 1 mM NaF, 1 mM Na_3_VO_4_ and 1 *μ*g/ml each of aprotinin, leupeptin and pepstatin). For phosphoprotein analysis, the cell pellets were lysed by sonication in 1X cell lysis buffer (50 mM Tris, 150 mM NaCl, 1mM EDTA, 1mM EGTA, 1% TritonX-100, 1X protease inhibitor cocktail, 1X phosphatase inhibitor cocktail, 1mM PMSF, 2mM Na_3_VO_4_, 100 nM okadoic acid, 0.5% sodium deoxycholate and 0.1% SDS). For co-immunoprecipitation, equal amounts of cell lysates were incubated with anti-CRM1 antibody (N-19, SC-7826, goat polyclonal, 1:500 dilution, Santa Cruz, USA) and the bound protein complexes were eluted and resolved on SDS-12%PAGE. The separated proteins were then transferred to a Hybond-P membrane (GE Healthcare, Sweden) and probed with monoclonal anti-GFP antibody (B-2, SC-9996, mouse monoclonal, 1:1000 dilution, Santa Cruz, USA). For assessing cyclin D1-cdk4 interaction, wild type or variants of Flag-GNL3L were transfected into HEK293 cells as described above. After 48 h of transfection, cells were lysed in 1X cell lysis buffer and the expression of GNL3L, endogenous cyclin D1 and cdk4 proteins were confirmed by western blotting. The expression levels of transfected proteins were normalized and co-immunoprecipitation was performed using anti-cyclin D1 antibody (556470, mouse monoclonal, 1:100 dilution, BD Biosciences, USA). The bound protein complexes were resolved on SDS-12% PAGE, transferred to Hybond-P membrane (GE Healthcare, Sweden) and probed with anti-cdk4 antibody (D9G3E, rabbit monoclonal, 1:1000 dilution, Cell Signaling Technology Inc., USA). Anti-Flag (F-3165, mouse monoclonal, 1:1500 dilution, Sigma- Aldrich, USA), anti-cyclin A (H-432,SC-751, rabbit polyclonal, 1:1000 dilution, Santa Cruz, USA), anti-cyclin B1 (H-433, SC-752, rabbit polyclonal, 1:1000 dilution, Santa Cruz, USA), anti-beta actin (A1978, mouse monoclonal,1:4000 dilution, Sigma-Aldrich, USA) and anti-GNL3L (ab94862, rabbit polyclonal, 1:500 dilution, Abcam, UK) antibodies were used for analyzing the expression of Flag-GNL3L or its variants and endogenous cyclin A, cylin B1, beta actin and GNL3L respectively. Monoclonal anti-Rb (4H1, mouse monoclonal, 1:500 dilution, Cell Signaling Technology Inc., USA) and anti-phospho-Rb (S780) (J146-35, mouse monoclonal, 1:500 dilution, BD Biosciences, USA) antibodies were used for analyzing the endogenous levels of total and phosphorylated retinoblastoma protein levels, respectively. The protein-antibody complexes were probed using horseradish peroxidase (HRP)-conjugated specific secondary antibodies (1:3000 dilution, Southern Biotechnology, USA) and detected using the Enhanced Chemi-luminescence Prime detection system (GE healthcare, Sweden).

### Fluorescence Microscopy

HeLa, COS-7 or HepG2 cells grown on chamber slides (BD Biosciences, USA) were infected with vTF7-3 prior to lipofectin mediated transfection of GNL3L expression plasmids. For determining the subcellular distribution of GNL3L, transfected cells were fixed using 3% (w/v) paraformaldehyde (for GFP-GNL3L) at 12–16 h post transfection or fixed with methanol (for Flag-GNL3L). Flag-GNL3L transfected cells were stained with polyclonal anti-Flag antibody (1:100 dilution, Sigma Aldrich, USA) followed by Alexa fluor 488 conjugated secondary antibody (1:1000 dilution, Molecular Probes, USA). The slides were mounted in mounting medium containing 4, 6-diamidino-2-phenylindole (DAPI) (Vector Laboratories, USA) to stain the nuclei. Nucleolin was stained with anti-nucleolin antibody (D4C70, rabbit monoclonal, 1:1000 dilution, Cell Signaling Technology Inc., USA) followed by Alexa fluor 594 conjugated secondary antibody (1:800 dilution, Molecular Probes, USA). Samples were then viewed either with Nikon TE2000 microscope (Nikon, Japan) or LSM710 laser scanning confocal microscope (Carl Zeiss, Germany) and image acquisition was performed using Image Pro-plus 4.5 software (Media Cybernetics, USA) and Zen 2009 software (Carl Zeiss, Germany), respectively.

### Cell Cycle Analysis

HEK293 and MCF-7 cells were transfected with expression vectors encoding wild type or variants of Flag-GNL3L in triplicates using lipofectamine (Invitrogen Life Technologies, USA). After 48 h of transfection, cells were lysed in hypertonic buffer containing propidium iodide (0.1% trisodium citrate, 0.1% NP40, 50μg/ml RNase, 45μg/ml propidium iodide) to obtain the nuclear fraction and the cell cycle pattern was analyzed by FACS CantoII (BD Biosciences, USA).

### Cell Synchronization

MCF-7 cells cultured on 12 well plates were transfected with expression plasmids encoding wild type or variants of Flag-GNL3L using lipofectamine 2000 (Invitrogen Life Technologies, USA). After 24 h of transfection, thymidine was added to the cells at a final concentration of 2mM (to block the cell cycle at G1/S boundary) and incubated at 37°C for another 24 h [29]. After the incubation, thymidine containing medium was removed, cells were washed thrice with 1X PBS, cultured in fresh DMEM and collected at various time points. Hypertonic buffer containing propidium iodide was used to obtain the nuclear fraction. The cell cycle patterns were analyzed by FACS Canto II (BD Biosciences, USA).

### Heterokaryon Assay

HeLa cells (1×10^5^) cultured on chamber slides (BD Biosciences, USA) were infected with vTF7-3 at a multiplicity of infection of 5 prior to lipofectin mediated transfection of wild type or mutants of GNL3L expression plasmids. After 16 h of transfection, cells were treated with DMEM containing 50μg/ml cycloheximide (CHX) for 4 h. Untransfected murine NIH-3T3 (1×10^4^) cells were then layered on top of the transfected HeLa cells and allowed to adhere for 4 h. The cells were then treated with 50μg/ml CHX for 1 h to inhibit *de novo* protein synthesis and cell fusion was induced by 50% wt/vol polyethylene glycol (PEG 3350, Sigma Aldrich, USA) in PBS for 2 min. CHX treatment was continued for additional 1 h and subsequently, the cells were fixed using 3% (w/v) paraformaldehyde. Nuclei were differentially stained with Hoechst 33342 (50μg/ml) (Sigma Aldrich, USA) for 10 min before being mounted using mounting medium (Vector Laboratories, USA).

### BrdU Incorporation Assay

The rate of cell proliferation was analyzed using BrdU Cell Proliferation Assay Kit (Cell Signaling Technology Inc., USA) according to manufacturer’s protocol. Briefly, HEK293 cells (1×10^5^) were cultured on a 96 well plate and transfected with wild type or variants of GNL3L. After 48 h of transfection, DMEM containing 1X BrdU was added to the cells and incubated for 4 h. Subsequently, cells were fixed for 30 min at room temperature and incubated with anti-BrdU primary antibody followed by HRP-conjugated secondary antibody. Finally, the cells were incubated with 3, 3’, 5, 5’-Tetramethylbenzidine (TMB) substrate and the absorbance was read at 450 nm using Microplate Reader (Bio-Rad Laboratories, USA).

### RT-qPCR Analysis

Total RNA was isolated from cells transfected with wild type or variants of GNL3L using TRIzol reagent (Invitrogen Life Technologies, USA). Reverse transcription was carried out using ImProm-II Reverse Transcription System (Promega Corporation, USA) according to manufacturer’s protocol. qPCR was carried out to quantitate mRNA expression levels in Realplex cycler (Eppendorf, Germany) using SYBR-Green mix (Roche, USA). All reactions were carried out in triplicates and the relative gene expression levels were analyzed using ∆Cτ values according to manufacturer’s instructions. The integrity of RNA was checked using beta actin primers and the mRNA expression levels of ectopically expressed GNL3L mutants were used as a reference for analyzing cyclin E1, cyclin A2 and E2F1 levels. The primers used for qPCR analysis are shown in S1 Table.

### Statistical Analysis

Statistical analysis was carried out using Graph Pad Prism 5.0 software. Error bars representing mean ± S.D are drawn from three independent transfections performed in parallel. Representative data from atleast three such independent experiments have been presented. The ‘p’ values indicative of statistical significance were obtained by performing student’s ‘t’ test (unpaired).

## Results

### GNL3L is a nucleo-cytoplasmic shuttling protein and its export from the nucleus is sensitive to Leptomycin B

**Fig 1:**
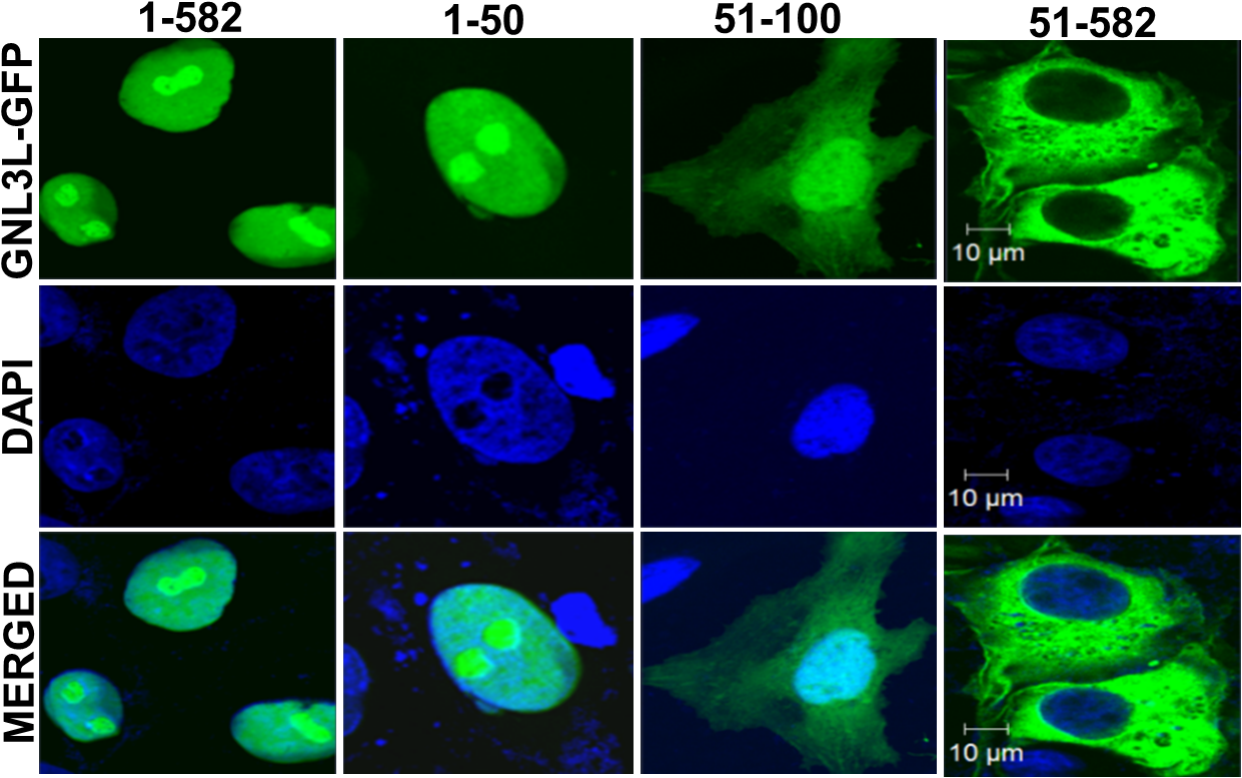
GNL3L localizes to distinct subcellular compartments. COS-7 cells were transfected with GFP tagged wild type or deletion mutants of GNL3L (GNL3L^1–50^, GNL3L^51–100^ and GNL3L^51–582^) and their sub-cellular distribution was analyzed using fluorescence microscopy. The scale bar represents 10 μm.

**Fig 2:**
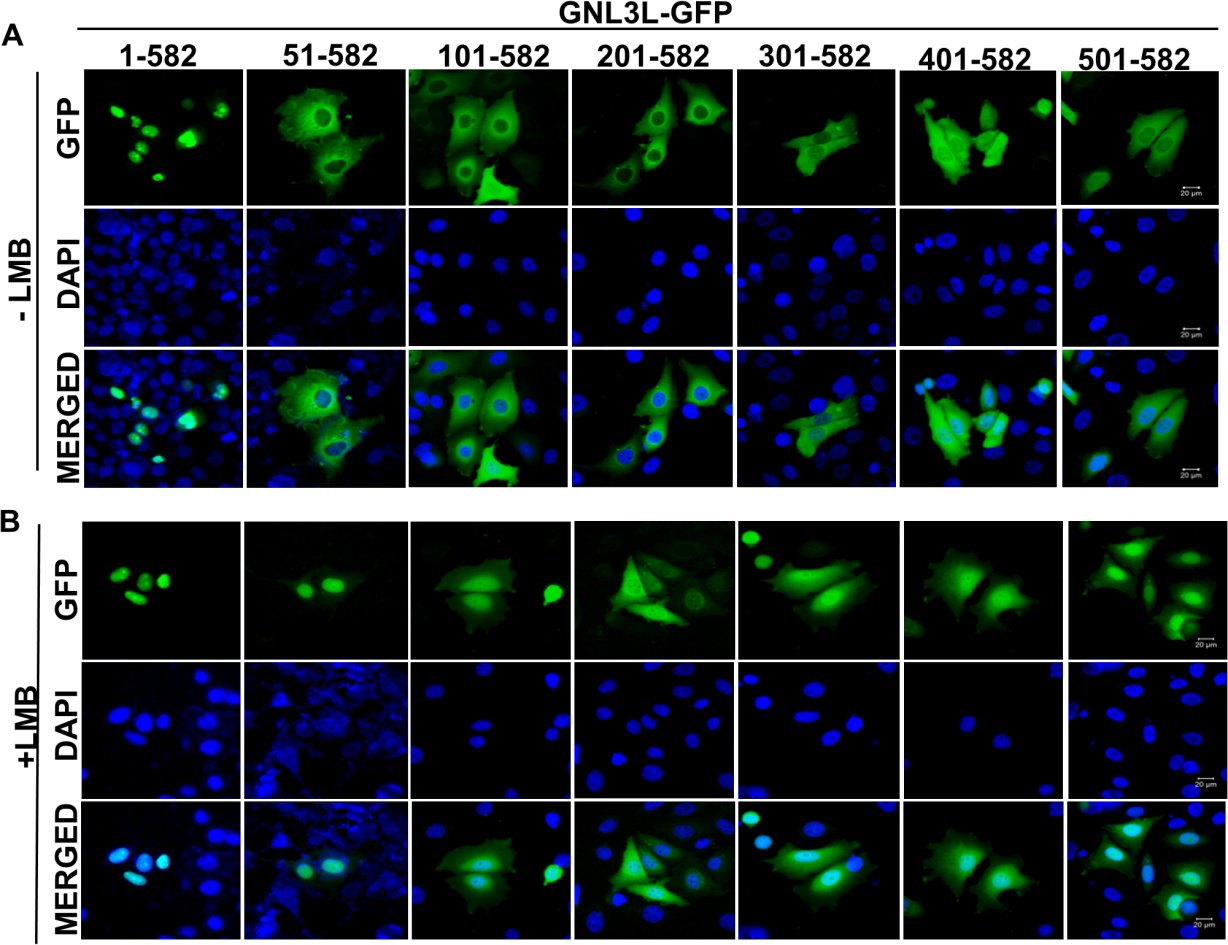
GNL3L shuttles between nucleus and cytoplasm and the export from the nucleus is sensitive to Leptomycin-B. HepG2 cells were transiently transfected with full length or indicated N-terminal deletion mutants of GNL3L and subcellular localization was determined in the presence (**B**) or the absence (**A**) of leptomycin-B (LMB). Nuclei were stained with DAPI. The scale bar represents 20μm.

GNL3L is localized predominantly in the nucleolus [9] and the depletion of intracellular GTP by mycophenolic acid (MPA), a GTP synthesis inhibitor, altered GNL3L subcellular distribution [9]. Comparison of GTP-binding abilities of wild type and G-domain variants of GNL3L with their subcellular localization patterns [9] (S1 Fig) suggests that GTP-binding is important for efficient nucleolar retention of GNL3L. These data led to the hypothesis that GNL3L could shuttle between nucleolus, nucleus and cytoplasm and GTP-binding may regulate this process. In order to define whether GNL3L is a nucleo-cytoplasmic shuttling protein, various deletion mutants of GNL3L were generated as GFP fusion proteins. Subcellular distribution of GNL3L mutants suggest that GNL3L^51–582^-GFP mutant protein localized in the cytoplasm despite having a functional nuclear localization signal (NLS) within amino acids 51–100 (Fig 1). This observation raises the possibility that a functional nuclear export signal (NES) resides towards the C-terminus of GNL3L and the observed cytoplasmic localization of GNL3L^51–582^ may be due to faster export kinetics. To further identify the minimal functional NES required for GNL3L nuclear export, systematic deletion analysis of GNL3L was performed. Wild type and the variants of GNL3L were transfected in HepG2 cells and their subcellular localization was determined by immunofluorescence analysis. Results in Fig 2A and S2 Fig clearly indicate that all the GNL3L mutant proteins were localized predominantly in the cytoplasm except the wild type protein (1–582), which was localized in the nucleolus. As expected, wild type GNL3L colocalized with nucleolin in the nucleolus (S2 Fig). Collectively, these results suggest the possibility that GNL3L may be shuttling between nucleus and cytoplasm by a signal-mediated process.

**Fig 3:**
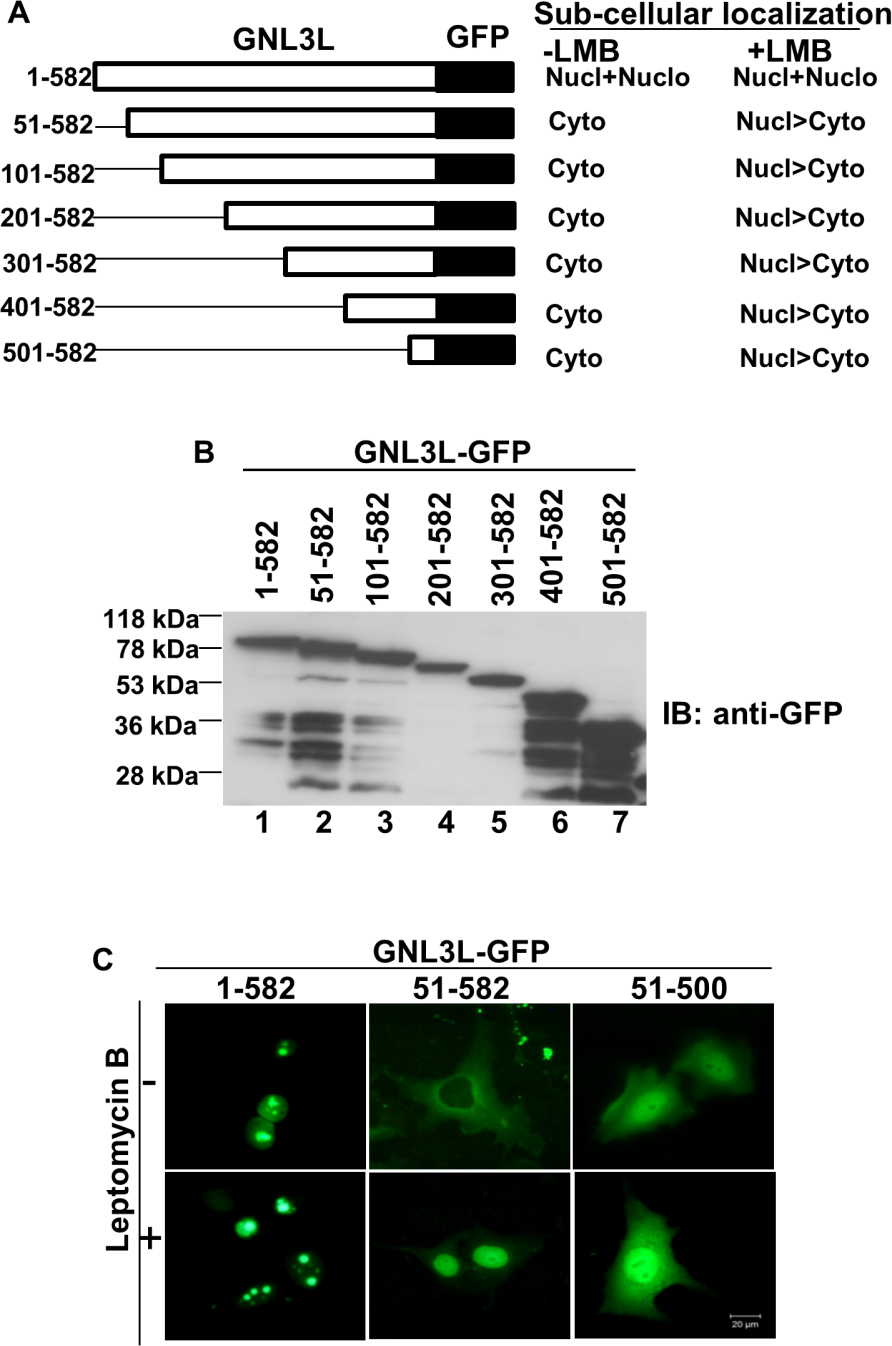
The C-terminal domain of GNL3L harbours a nuclear export signal. (**A**)Expression of wild type and indicated deletion mutants of GNL3L was determined by western blot using anti-GFP antibody. The indicated variants of GNL3L were expressed correct sized polypeptides. **(B)** Schematic diagram of GNL3L deletion mutants and summary of their subcellular distribution. (**C**) Cells expressing full length and deletion mutants of GNL3L were treated with (+LMB) or without (-LMB) and subcellular distributions were analyzed by fluorescence microscopy.

NESs are known to be rich in leucine or other hydrophobic residues and the consensus motif may correspond to Φ-X_2–3_-Φ-X_2–3_- Φ-X-Φ pattern, where Φ denotes hydrophobic residues such as leucine, isoleucine, valine or phenyl alanine. Primary sequence analysis of GNL3L suggests the presence of a stretch of hydrophobic residues matching this consensus sequence within its C-terminal domain. CRM1 (chromosome maintenance region 1/Exportin-1/Xpo1), is the major nuclear export receptor that recognizes and binds to such leucine-rich signals and transports the cargo across the nuclear pore complex (NPC) in a Ran-GTP dependent manner [30]. Leptomycin-B (LMB) is a well-known inhibitor of CRM1-mediated nuclear export [18]. It binds and alkylates Cysteine 529 residue of CRM1, thereby blocking its interaction with NES bearing cargo. Hence, we first analyzed whether GNL3L is exported from the nucleus by a CRM1 dependent pathway. Towards this end, HepG2 cells transfected with the wild type or different deletion mutants of GNL3L were treated with LMB. Interestingly, increased nuclear localization (excluded from the nucleolus) was observed for all the GNL3L mutant proteins in the presence of LMB (Fig 2B). These results suggest that GNL3L is exported from the nucleus by CRM1 dependent pathway and the export process is sensitive to LMB. The nucleolar localization of full length GNL3L was not altered when cells were treated with LMB (Fig 2B). Quantitation of the subcellular distribution of wild type and indicated variants of GNL3L was performed in three independent experiments and the results clearly indicate that majority of GNL3L mutant proteins redistributed to the nuclear compartment upon LMB treatment (S3 Fig). Expression and integrity of GNL3L mutants was determined by western blot analysis (Fig 3A) and DNA sequencing. Thus, the subcellular localization patterns of all indicated mutants of GNL3L in the presence or absence of LMB (Figs 2A, 2B and 3B) suggest that the carboxyl terminal domain (amino acids 501–582) harbors a signal required for CRM1 dependent export of GNL3L from the nucleus. Localization patterns of the mutant polypeptides remained similar in the cell lines tested suggesting that the transport mechanism of GNL3L is conserved across various cell types.

We next tested the importance of C-terminal domain on GNL3L export from the nucleus by generating additional C-terminal deletion mutants as GFP fusion proteins. Wild type and the mutants of GNL3L were transfected into COS-7 cells and treated with LMB as described above. Immunofluorescence analysis indicates that the mutant lacking the C-terminal domain (GNL3L^51–500^-GFP), showed a diffused localization (Fig 3C). In contrast, the GNL3L mutant containing the C-terminal 82 amino acids (GNL3L^51–582^-GFP) relocated to the nuclear compartment after LMB treatment (Fig 3C, lower panel). Together, these data suggest that a functional NES resides between amino acids 501 to 582 in the C-terminus and is sufficient for the export of GNL3L between nucleus and cytoplasm.

**Fig 4:**
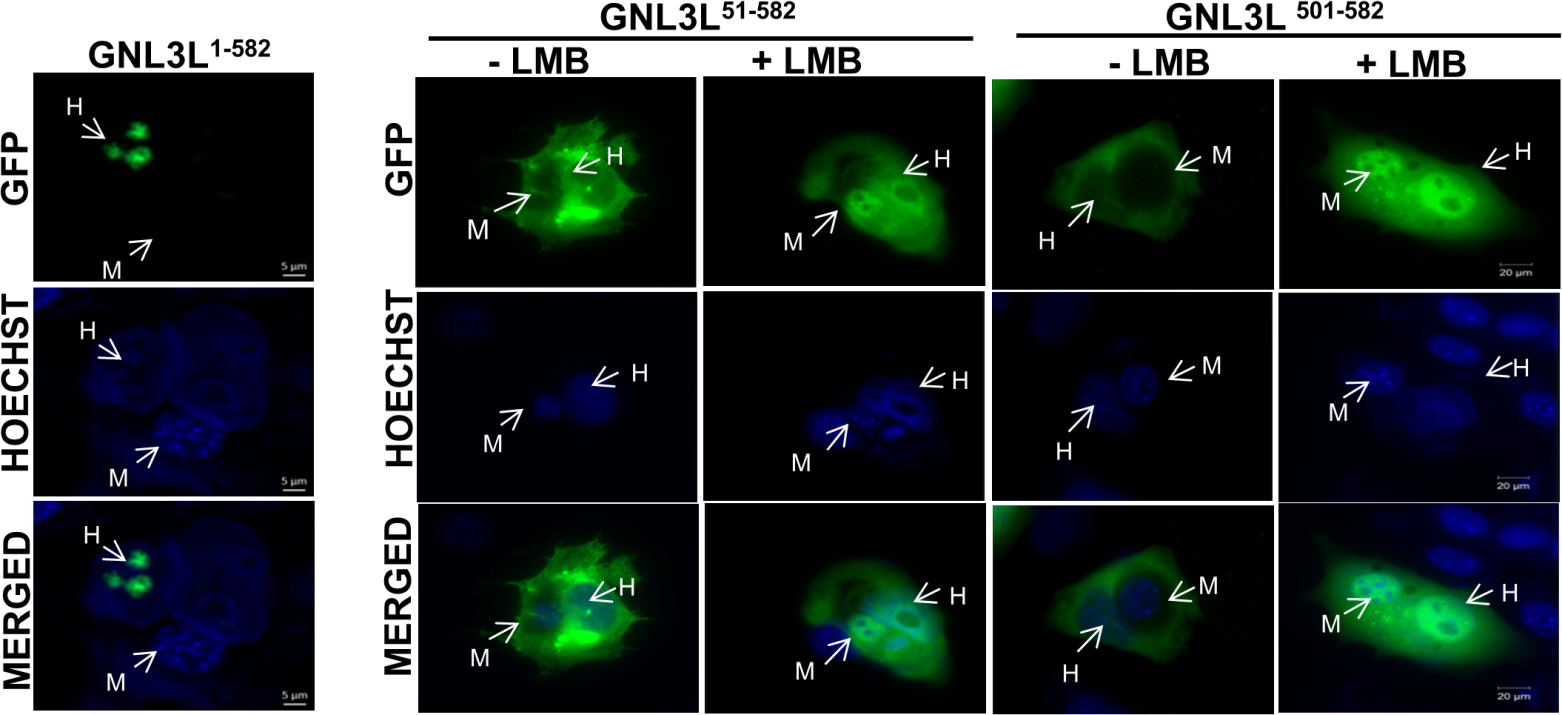
GNL3L is a nucleo-cytoplasmic shuttling protein. Heterokaryon assay was performed by transfecting GNL3L^1–582^-GFP, GNL3L^51–582^-GFP or GNL3L^501–582^-GFP into HeLa cells and fusing with NIH3T3 cells as described in Materials and Methods. The punctate pattern of murine nucleus (‘M’) upon staining with Hoechst 33342 distinguished it from the human HeLa nucleus (‘H’). In the presence of LMB, the nuclear export of GNL3L^51–582^-GFP or GNL3L^501–582^-GFP was prevented and the protein was localized in both HeLa and NIH3T3 nucleus.

In order to further confirm the nucleo-cytoplasmic shuttling of GNL3L, heterokaryon assay was performed using HeLa cells transiently transfected with wild type GNL3L or GNL3L^51–582^-GFP or GNL3L^501–582^-GFP. After 24 h, transfected HeLa cells were fused with untransfected NIH-3T3 cells as described in Materials and Methods. If GNL3L is actively exported to the cytoplasm by a signal-mediated energy dependent process, GNL3L signal will appear both in the HeLa (human) and NIH-3T3 (mouse) nuclei. Mouse nucleus can readily be distinguished from HeLa nuclei by the punctate fluorescence pattern upon Hoechst 33342 staining. Both GNL3L^51–582^-GFP and GNL3L^501–582^-GFP were localized in the cytoplasm of the heterokaryon in the absence of LMB (Fig 4). Interestingly, GNL3L^51–582^-GFP and GNL3L^501–582^-GFP specific signals were observed in the nuclei of both HeLa and NIH-3T3 cells upon LMB treatment (Fig 4). Surprisingly, the retention of GNL3L^1–582^-GFP in the HeLa nucleolus was not altered in the presence or absence of LMB treatment suggesting the possibility that GNL3L may interact with other nucleolar/ribosomal components through its N-terminal NoLS to be retained in the nucleolus. Together, these results provide evidence that GNL3L is a nucleo-cytoplasmic shuttling protein and the C-terminal domain is necessary and sufficient for the export of GNL3L from the nucleus by a signal-mediated process.

### GNL3L interacts with Nuclear Export Receptor CRM1

**Fig 5:**
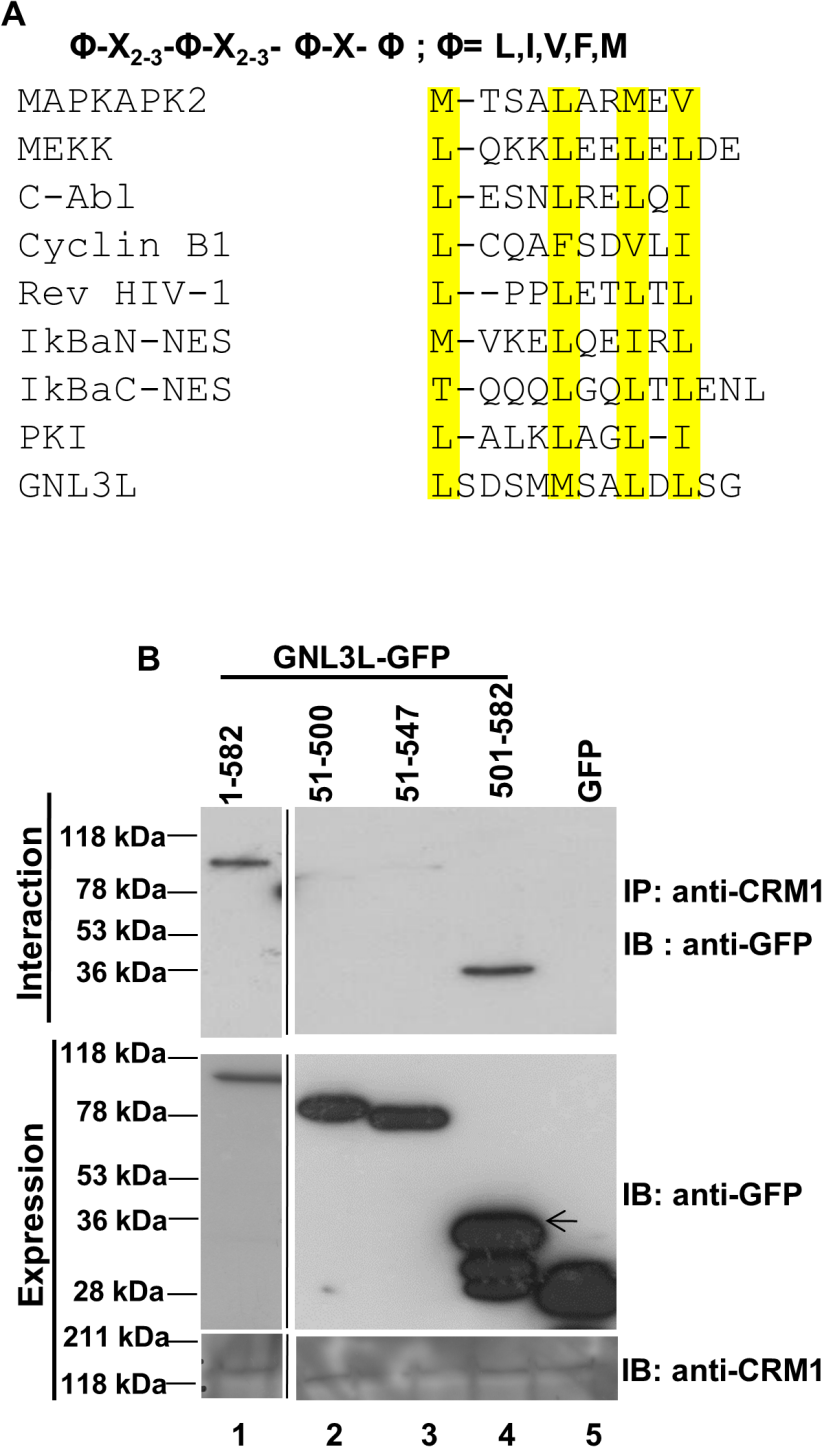
GNL3L interacts with the export receptor, CRM1 via C-terminal domain. (**A**) Comparison of GNL3L Nuclear Export Signals (NES) with known NESs from various nucleo-cytoplasmic shuttling proteins. Conserved hydrophobic residues are highlighted in yellow colour. (**B**) Wild type or indicated variants of GNL3L were transiently transfected into HeLa cells and the cell lysates were subjected to co-immunoprecipitation with anti-CRM1 antibody followed by western blot using anti-GFP antibody (Top panel). The expression of GNL3L and CRM1 (endogenous) was confirmed prior to immunoprecipitation by western blot using anti-GFP (lower panel) and anti-CRM1 antibodies (bottom panel).

CRM1 recognizes cargoes with hydrophobic amino acid-rich nuclear export signals. The consensus sequence required for CRM1 recognition and a comparison of GNL3L nuclear export signals with the NESs from diverse classes of nucleo-cytoplasmic shuttling proteins have been summarized in Fig 5A. Having demonstrated that GNL3L is a nucleo-cytoplasmic shuttling protein and the nuclear export is sensitive to LMB, we next tested whether GNL3L physically interacts with export receptor, CRM1 by co-immunoprecipitation assay. Wild type and various deletion mutants of GNL3L-GFP were transiently transfected in HeLa cells and expression of these fusion proteins was determined by western blot analysis using anti-GFP antibody (Fig 5B; middle panel). Endogenous expression of CRM1 was analyzed using anti-CRM1 antibody (Fig 3B; lower panel). Equal amounts of cell lysates were used in co-immunoprecipitation assay with anti-CRM1 antibodies followed by western blot analysis using anti-GFP antibody. Interestingly, results in Fig 5B indicate that GNL3L^501–582^ interacts with CRM1 (Upper panel; lane 4) similar to wild type GNL3L (Upper panel; lane 1). In contrast, GNL3L^51–500^ and GNL3L^51–547^ failed to interact with CRM1 (Fig 5B Upper panel; lane 2 and 3). GFP was used as a negative control to determine the integrity of the assay. Collectively, these data suggest that GNL3L specifically interacts with CRM1 receptor and confirm that the NES present in the C-terminal domain of GNL3L is critical for this interaction as well as its export from the nucleus. It is worth noting that despite the strong interaction of GNL3L with CRM1 in a co-immunoprecipitation assay, we failed to observe the export of GNL3L wild type protein from HeLa nucleus in the heterokaryon assay. This may be due to its interaction with nucleolar proteins or other nucleolar structural components through its N-terminal NoLS.

**Fig 6:**
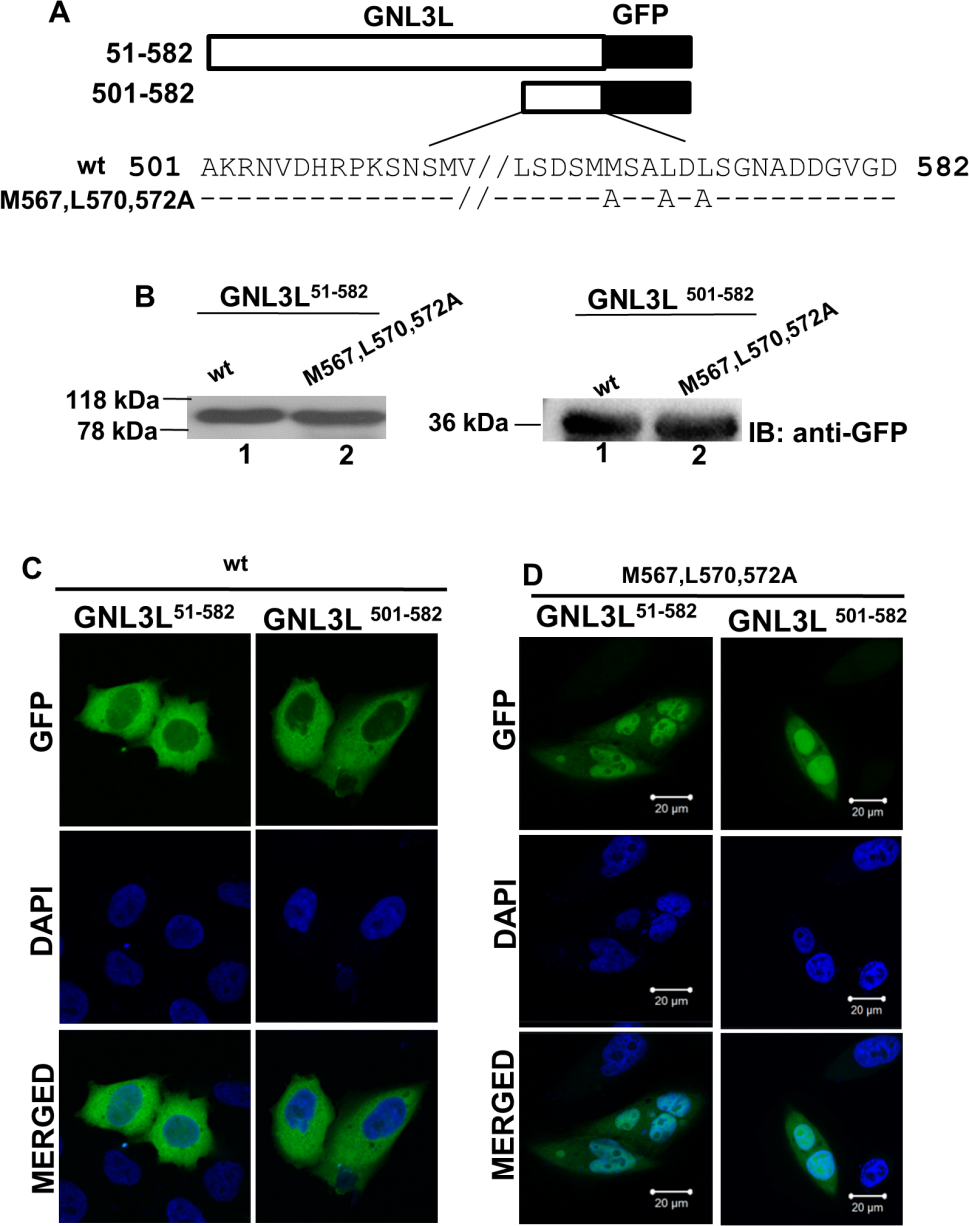
The hydrophobic residues within the C-terminal domain of GNL3L constitute a functional nuclear export signal. (**A**) Indicated variants of GNL3L ^51–582^-GFP and GNL3L ^501–582^-GFP were generated using QuickChange site-directed mutagenesis as described in Materials and Methods. (**B**) All the indicated GNL3L mutants were expressed correct size polypeptides as evident from the western blot analysis using anti-GFP antibody. HeLa cells were transfected with wild type GNL3L ^51–582^-GFP or GNL3L ^501–582^-GFP (**C**) and their variants (**D**). Transfected cells were fixed in 3% paraformaldehyde and nuclei were stained using DAPI. Subcellular localization GNL3L variants were analyzed using confocal microscope. The scale bar represents 20 μm.

Immunofluorescence studies, protein-protein interaction assays and primary sequence analysis suggest that the amino acids 501–582 in the C-terminal domain of GNL3L may contain the functional nuclear export signal. To determine the critical residues that are required for GNL3L export from the nucleus, nuclear export mutants were generated by exchanging the conserved residues (Met567, Leu570 and 572 to Ala) pertaining to the consensus NES recognized by CRM1 using GNL3L^51–582^-GFP and GNL3L^501–582^-GFP as templates (Fig 6A). All the indicated GNL3L variants were transfected into HeLa cells and their expression was confirmed by western blot analysis. Results in Fig 6B suggest that the replacement of conserved hydrophobic residues did not alter the expression of mutant GNL3L proteins. GNL3L mutants were then transfected into HeLa cells and their subcellular localization was determined as described in Materials and Methods. Immunofluorescence analysis revealed that the GNL3L^51–582^ and GNL3L^501–582^ mutant proteins showed predominant nuclear localization compared to the cytoplasmic localization of wild type GNL3L^51–582^ and GNL3L^501–582^ (Fig 6C and 6D). These results clearly suggest that the conserved hydrophobic residues in the C-terminal domain constitute a functional NES for GNL3L.

### Nuclear localization of GNL3L is important for ‘S’ phase progression of cell cycle

**Fig 7:**
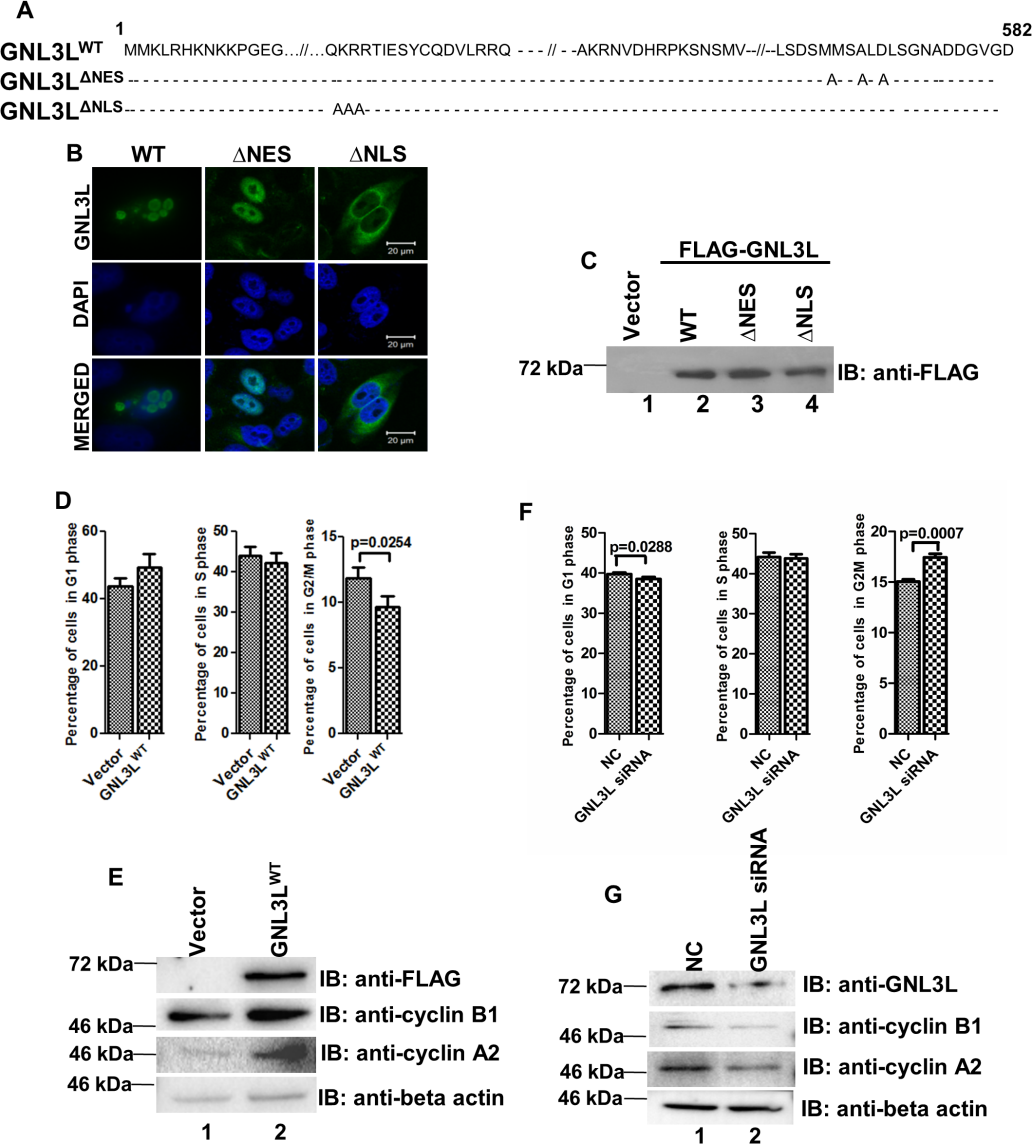
GNL3L modulates ‘G2/M’ phase progression. (**A**) Schematic representation of wild type, nuclear import (GNL3L^ΔNLS^) and nuclear export (GNL3L^ΔNES^) defective mutants of GNL3L. (**B**) HeLa cells were transfected with Flag-tagged GNL3L^WT^, GNL3L^ΔNES^ and GNL3L^ΔNLS^ expression plasmids and the subcellular localization was analyzed using confocal microscopy. The scale bar represents 20μm. (**C**) HEK293 cells were transfected with Flag-tagged GNL3L^WT^, GNL3L^ΔNES^ and GNL3L^ΔNLS^ and the expression was determined by western blot analysis using anti-Flag antibody. **(D)** Ectopic expression of GNL3L results in decreased accumulation of ‘G2/M’ population. GNL3L^WT^ was overexpressed in HEK293 cells and the asynchronous cell cycle pattern was analyzed using flow cytometry as described in Materials and Methods. **(E)** Western blot was performed to analyze the protein levels of endogenous cyclins A2 and B1 upon GNL3L^WT^ expression in HEK293 cells. Beta actin served as loading control. **(F)** GNL3L knockdown leads to increased accumulation of cells in G2/M phase of the cell cycle. Transient knockdown of GNL3L was performed in HEK293 cells using specific siRNA and the cell cycle profile was analyzed using flow cytometry. (NC: Negative control). **(G)** Endogenous cyclins A2 and B1 levels were analyzed using western blot upon GNL3L knockdown in HEK293.

To understand the functional consequences of differential subcellular distribution pattern of GNL3L, variants of GNL3L were generated by site-directed mutagenesis using Flag-GNL3L^1–582^ as template (Fig 7A). Wild type GNL3L^1–582^ (GNL3L^WT^) was nucleolar in localization whereas GNL3L^1–582 (M567, L570, 572A)^ (hereafter referred as GNL3L^∆NES^) exhibited nuclear localization (excluded from nucleolus) and GNL3L^1–582 (K81, R82, 83A)^ (hereafter referred as GNL3L^∆NLS^) localized to the cytoplasm (Fig 7B). Subcellular localization analysis indicated that the Flag tag did not have any effect on the localization of these fusion proteins. Western blot analysis using anti-Flag antibody indicated that all the GNL3L variants were expressed as correctly sized polypeptides (Fig 7C).

In order to understand the role of wild type GNL3L on cell cycle progression, GNL3L^WT^ was overexpressed in HEK293 cells and cell cycle analysis was performed. A significant decrease in the ‘G2/M’ population (p = 0.0254) was observed upon ectopic expression of GNL3L^WT (^ Fig 7D). The levels of endogenous cyclins A2 and B1, which are known to regulate ‘G2/M’ transition and progression, were also found to be upregulated (Fig 7E). Interestingly, transient knockdown of GNL3L resulted in significantly higher (p = 0.0007) accumulation of cells in the ‘G2/M’ phase (Fig 7F), which is consistent with a recent report [6]. In addition, decreased ‘G1’ phase population was also noted in GNL3L depleted cells. The observed ‘G2/M’ arrest may be due to the down regulation of cyclin A2 and cyclin B1 levels upon GNL3L knockdown (Fig 7G). Together, these results provided evidence that GNL3L^WT^ may play a critical role in ‘G2/M’ phase progression.

**Fig 8:**
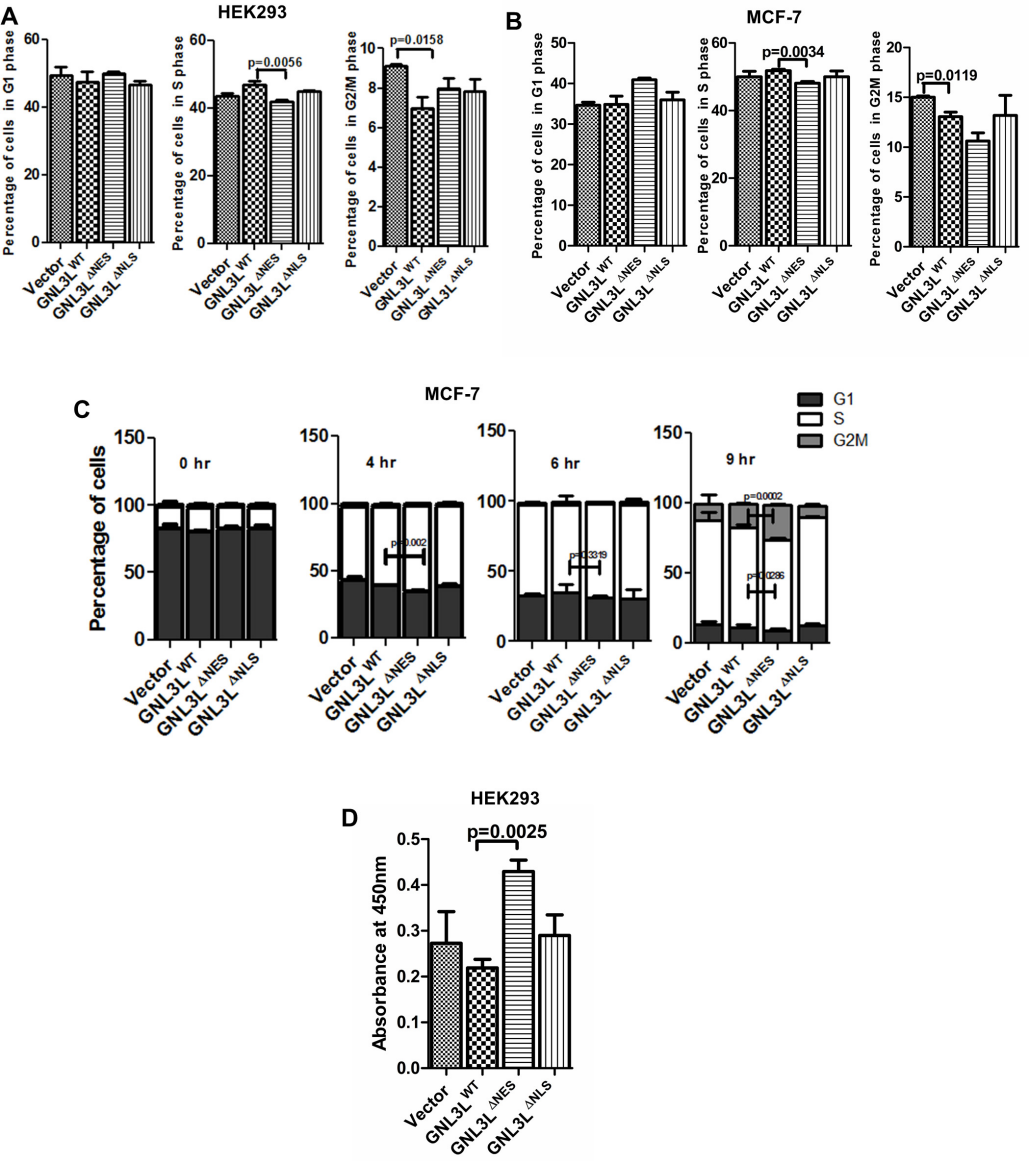
Nuclear localization of GNL3L promotes ‘S’ phase progression. HEK293 (**A**) and MCF-7 (**B**) cells were transfected with wild type or indicated variants of GNL3L and the asynchronous cell cycle profiles were analyzed. (**C**) MCF-7 cells transfected with wild type or variants of GNL3L were synchronized by single thymidine block as described in Materials and Methods. Cell cycle profiles were analyzed at 0, 4, 6 and 9 h post thymidine release by flow cytometry. Propidium Iodide (PI) was used to label the nuclei. (**D**) HEK293 cells were transfected with wild type or indicated variants of GNL3L and the DNA synthesis was measured by incubating the cells with 1X BrdU for 4 h followed by staining with anti-BrdU primary antibody and HRP-conjugated secondary antibody. Stained cells were incubated with TMB substrate and the absorbance was read at 450 nm.

Towards analyzing the effect of differential localization of GNL3L on cell cycle progression, GNL3L^WT^, GNL3L^∆NES^ and GNL3L^∆NLS^ were overexpressed in HEK293 cells and the cell cycle profiles were analyzed. The ectopic expression of GNL3L^∆NES^ resulted in a significant decrease in the percentage of cells in ‘S’ phase (p = 0.0056) as compared to GNL3L^WT^ and GNL3L^∆NLS^, suggesting that retention of GNL3L in the nuclear compartment (defective nuclear export) may be critical for ‘S’ phase progression (Fig 8A). In order to test whether the effect of differential distribution of GNL3L is similar in other cell types, we ectopically expressed all the indicated GNL3L variants in breast cancer cell line, MCF-7 (harbours wild type p53 and Rb [31]). In consistence with the data obtained from HEK293 cells, GNL3L^∆NES^ overexpression resulted in significant decrease in the percentage of cells in ‘S’ phase (p = 0.0034) as compared to GNL3L^WT^ (Fig 8B). The distinct cell cycle profiles upon ectopic expression of wild type and variants of GNL3L suggest the specificity of GNL3L function during cell proliferation.

In order to define the mechanism of GNL3L mediated ‘S’ phase regulation, MCF-7 cells expressing GNL3L^WT^, GNL3L^∆NES^ and GNL3L^∆NLS^ were synchronised at ‘G1/S’ boundary by single thymidine block and cell cycle analysis was performed at various time points after the release from thymidine block as described in Materials and Methods. Results in Fig 8C suggest that significantly higher amount of cells entered ‘S’ phase at the end of 4 h (p = 0.002) post thymidine release in GNL3L^∆NES^ compared to GNL3L^WT^ expressing cells. Also, GNL3L^∆NES^ transfected cells progressed faster through the ‘S’ phase (p = 0.0286) and entered ‘G2/M’ phase (p = 0.0002) at the end of 9 h in comparison with GNL3L^WT^. To further understand whether GNL3L^∆NES^ mediated ‘S’ phase progression is due to rapid DNA synthesis, BrdU incorporation assay was performed in cells transiently transfected with GNL3L^WT^, GNL3L^∆NES^ and GNL3L^∆NLS^. Results in Fig 8D indicated that the BrdU incorporation in cells expressing GNL3L^∆NES^ (p = 0.0025) was higher than GNL3L^WT^ and GNL3L^∆NLS^, which positively correlated with increased DNA synthesis. Collectively, these data suggest that nuclear retention of GNL3L may be important to promote ‘S’ phase progression during cell division cycle.

### Ectopic expression of GNL3L^∆NES^ upregulates E2F1, Cyclin A2 and Cyclin E1 expression to promote ‘S’ phase progression during cell cycle

**Fig 9:**
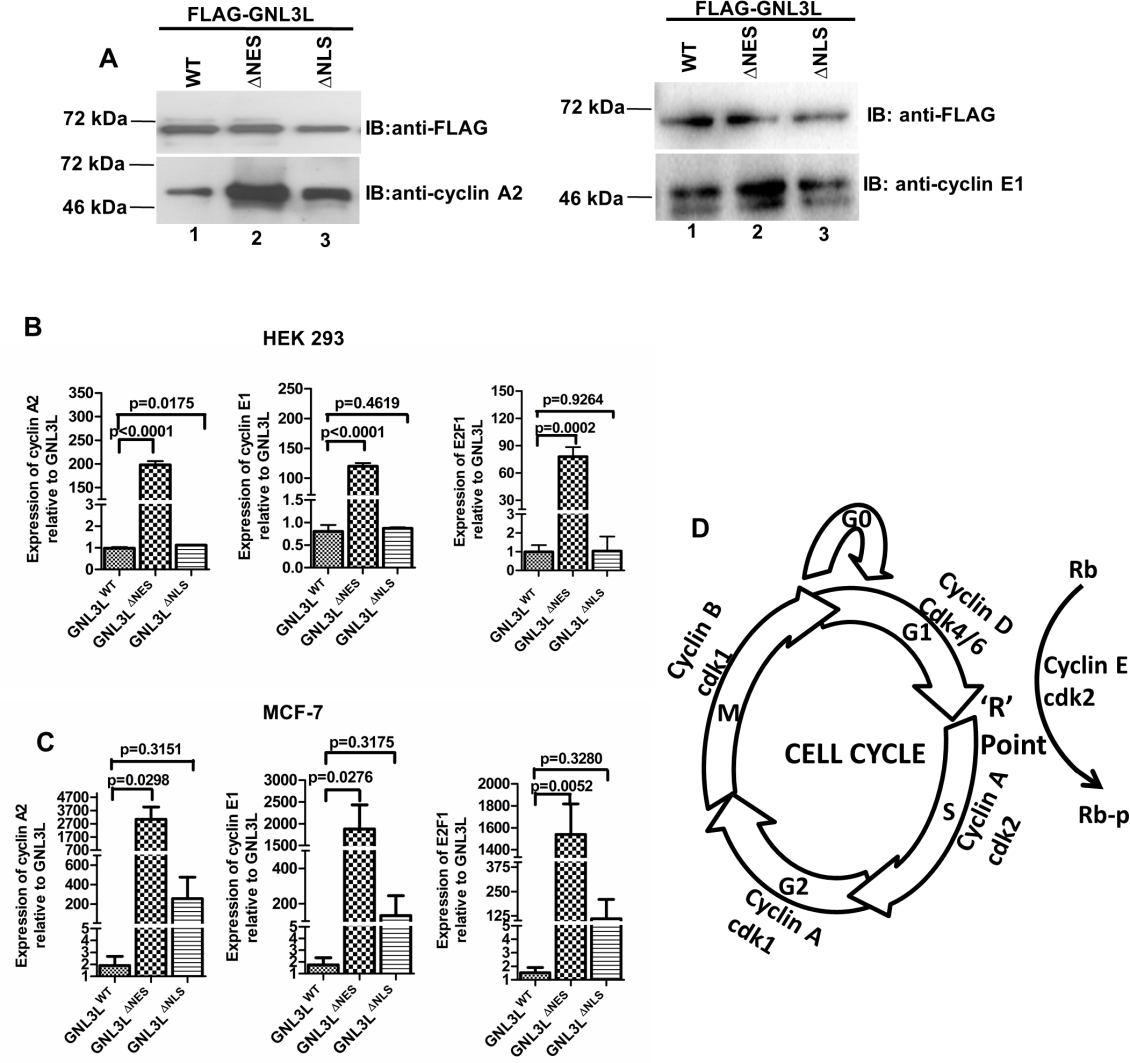
Nuclear localization of GNL3L modulates the levels of E2F1, cyclins A2 and E1 to promote ‘S’ phase progression. (**A**) Ectopic expression of GNL3L^ΔNES^ upregulates endogenous cyclin A2 and cyclin E1 protein levels. HEK293 cells were transfected with wild type or indicated variants of GNL3L and the cyclin levels were analyzed by western blot using anti-cyclin A and anti-cyclin E1 antibodies. Real-time qPCR analysis suggests that ectopic expression of GNL3L^ΔNES^ upregulates E2F1, cyclin A2 and cyclin E1 transcription in HEK293 **(B)** and MCF-7 **(C)** cells. (**D**) Schematic diagram showing the cyclins and cyclin dependent kinases mediated cell cycle regulation.

Towards defining the molecular mechanism by which GNL3L promotes ‘S’ phase progression, we first analyzed the status of cyclins that regulate the ‘S’ phase transition, particularly cyclins A2 and E1. Indicated variants of GNL3L were transiently expressed in HEK293 cells and equal amounts of cell lysates were resolved in SDS-12%PAGE followed by western blot using anti-Flag (for GNL3L), anti-cyclin A2 and anti-cyclin E1 antibodies. Interestingly, consistent with faster ‘S’ phase progression, a relatively higher amount of cyclins A2 and E1 was observed in GNL3L^∆NES^ expressing cells as compared to GNL3L^WT^ and GNL3L^∆NLS^ (Fig 9A). To further understand whether the observed increased levels of these cyclins were due to stimulation of their transcription, real-time quantitative PCR (RT-qPCR) was performed using RNA isolated from GNL3L^WT^, GNL3L^∆NES^ and GNL3L^∆NLS^ expressing cells using cyclin A2 and cyclin E1 specific primers (S1 Table). For normalization, mRNA levels of overexpressed GNL3L variants were determined using specific primers such that they will amplify only the ectopically expressed but not the endogenous GNL3L. Equal amounts of cDNA were used to amplify the targets and the cDNA integrity was checked using beta-actin primers. In accordance with higher protein levels, significantly higher levels of cyclins A2 and E1 mRNA were noticed in GNL3L^∆NES^ compared to GNL3L^WT^ and GNL3L^∆NLS^ overexpressing cells (Fig 9B). Since cyclins A2 and E1 are transcriptional targets for the transcription factor E2F1 [32], relative expression of E2F1 was analyzed upon GNL3L expression. Results in Fig 9B indicate that there was a significant increase in E2F1 mRNA levels in GNL3L^∆NES^ expressing cells as compared to GNL3L^WT^ and GNL3L^∆NLS^. Similar results were also obtained in MCF-7 cells (Fig 9C). The difference in the relative expression of E2F1, cyclins A2 and E1 between HEK293 and MCF-7 may be due to the differential transfection efficiencies or additional unexplored factors that play a role in E2F1 mediated transactivation.

**Fig 10:**
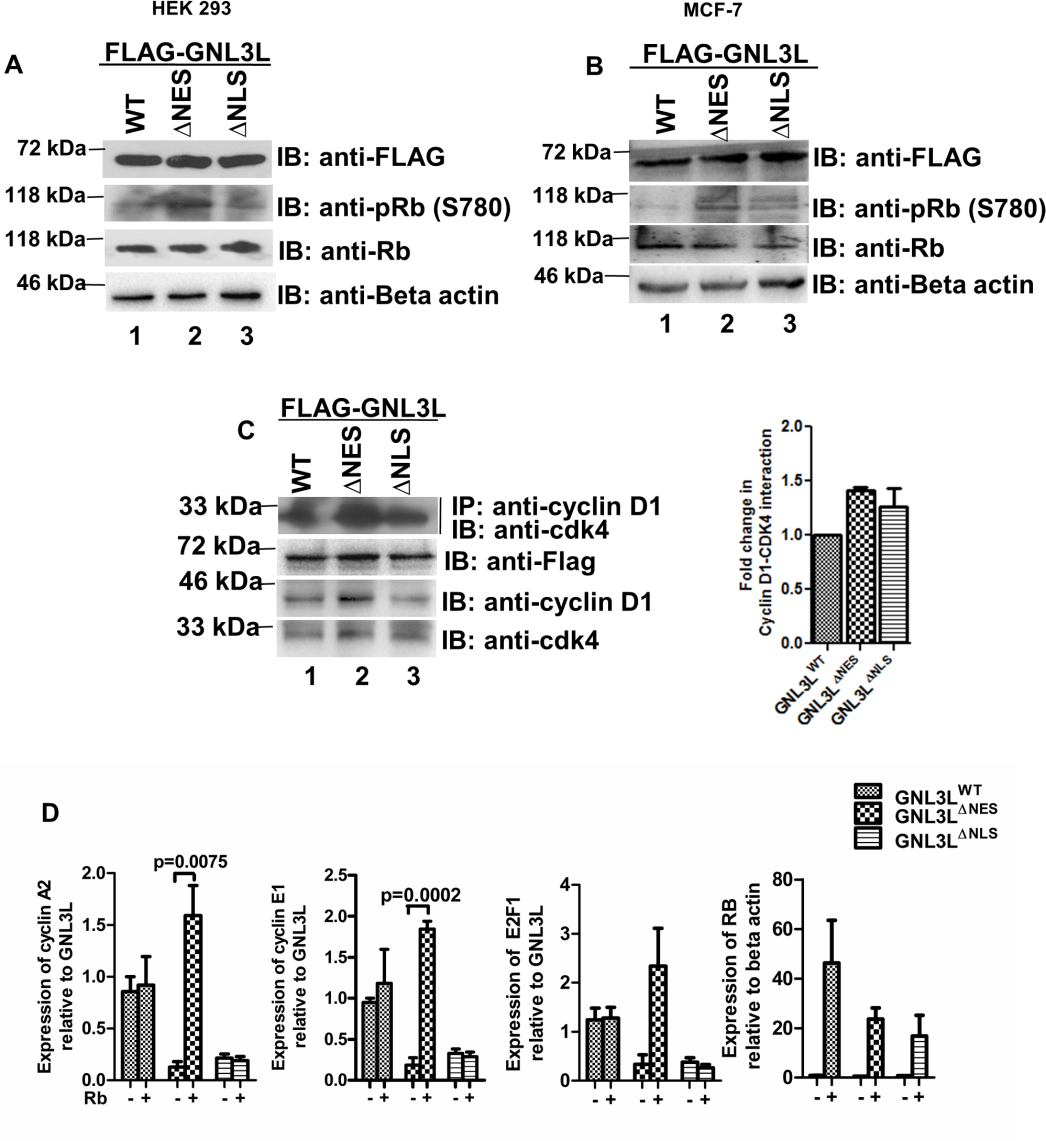
Nuclear localization of GNL3L modulates Rb-E2F1 pathway. Ectopic expression of GNL3L^ΔNES^ leads to hyperphosphorylation of Retinoblastoma protein (Rb) at serine 780. HEK293 **(A)** and MCF-7 **(B)** cells were transfected with wild type or variants of GNL3L. Anti-phospho Rb (S780) antibody and anti-Rb antibodies were used to determine the status of Rb phosphorylation and total Rb protein levels, respectively, by western blot analysis. Beta actin served as loading control. (**C**) GNL3L^ΔNES^ expression leads to increased association between cyclin D1 and cdk4. HEK293 cell lysates containing wild type or variants of GNL3L were subjected to co-immunoprecipitation with anti-cyclin D1 antibody followed by western blot using anti-cdk4 antibody. Expression levels of the indicated variants of GNL3L were normalized to that of GNL3L^WT^ and used as the reference for these analyses. **(D)** GNL3L^ΔNES^ displays Rb dependency in the upregulation of E2F1, cyclins A2 and E1 levels. The Rb null cell line, Hep3B was transfected with wild type or variants GNL3L, alone or in combination with Rb expression plasmid. RT-qPCR was performed for the respective genes using specific primers (S1 Table).

E2F1 activity is normally repressed until the ‘R’ point of G1-S transition by binding to hypophosphorylated Rb protein (Fig 9D) [33]. Cyclin D1-cdk4/6 phosphorylates Rb at serine 780 in order to override the ‘R’ point [34]. To further define whether the expression of GNL3L^∆NES^ promotes Rb phosphorylation at Serine 780 (pRb^S780^), which in-turn releases E2F1 from hypophosphorylated Rb-E2F1 inhibitor complex to transactivate its target genes, the phosphorylation status of Rb was determined upon ectopic expression of GNL3L^WT^, GNL3L^∆NES^ and GNL3L^∆NLS^. Interestingly, level of pRb^S780^ was higher in GNL3L^∆NES^ expressing cells compared to GNL3L^WT^ and GNL3L^∆NLS^ without altering total Rb protein level (Fig 10A). Similar status of Rb phosphorylation was also observed in MCF-7 cells (Fig 10B). To further understand the mechanism of GNL3L induced ‘S’ phase progression, cyclin D1-cdk4 complex formation was assessed in cells expressing GNL3L^WT^, GNL3L^∆NES^ and GNL3L^∆NLS^. Ectopic expression of GNL3L^∆NES^ led to a modest increase in cyclin D1-cdk4 association as compared to GNL3L^WT^ and GNL3L^∆NLS^ in HEK293 cells. (Fig 10C).

To further confirm the involvement of Rb in GNL3L mediated cell cycle regulation, we analyzed the levels of E2F1, cyclins A2 and E1 in Rb null cell line, Hep3B [35]. Co-expression of Rb with GNL3L^∆NES^ but not with GNL3L^WT^ or GNL3L^∆NLS^ resulted in increased levels of E2F1, cyclins A2 and E1 (Fig 10D). It is worth noting that GNL3L^WT^ expression led to an increase in E2F1, cyclins A2 and E1 levels as compared to GNL3L^∆NES^ even in the absence of Rb. These data suggest the existence of an alternative pathway of E2F1 regulation by GNL3L in Rb null cells. Taken together, our results suggest that GNL3L may enhance the association of cyclin D1 and cdk4 to phosphorylate Rb at Serine 780, which in-turn upregulates E2F1 target gene expression to promote ‘S’ phase progression.

## Discussion

**Fig 11:**
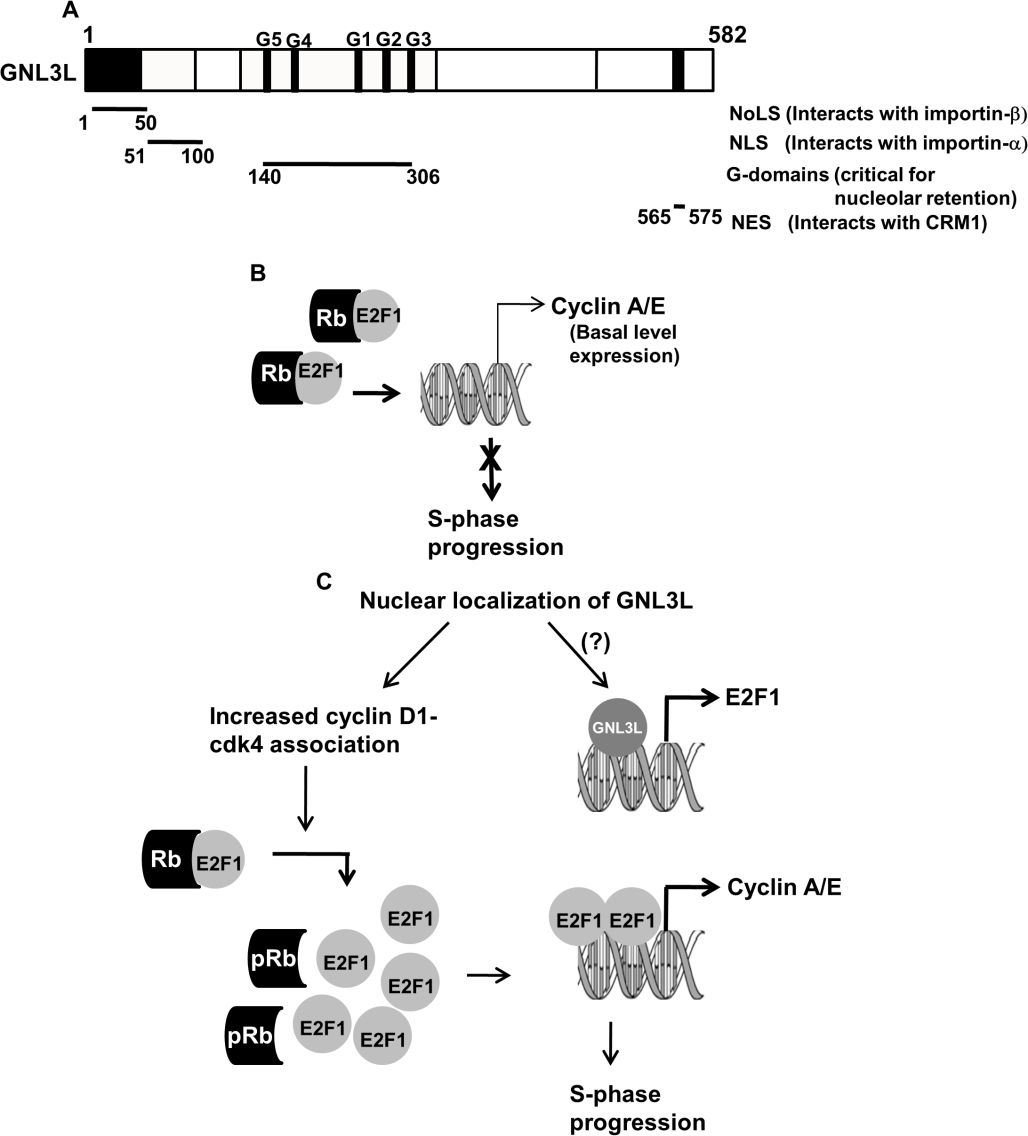
Proposed model for GNL3L function during cell proliferation. (**A**) GNL3L harbors multiple functional domains. Amino terminus of GNL3L encodes a functional nucleolar targeting signal (NoLS; amino acids 1–50) and a nuclear localization signal (NLS; amino acids 51–100). A functional nuclear export signal is mapped towards the carboxyl terminal domain (501–582) and the combination of mutagenesis and subcellular localization studies suggest that residues M567, L570 and 572 are critical for GNL3L export from the nucleus. (**B**) Interaction of Rb with E2F1 is essential to regulate cell proliferation by controlling E2F1 transcriptional activity. (**C**) The binding of Retinoblastoma protein (Rb) with E2F1 transcription factor inhibits transcription of S-phase regulatory genes as cyclin A2 and cyclin E1. Nuclear localization of GNL3L leads to phosphorylation of Rb at S780, which is critical to release E2F1 to activate transcription of cyclin A and cyclin E, resulting in ‘S’ phase progression. These data suggest the possibility that nuclear localization of GNL3L promotes ‘S’ phase progression during cell proliferation by modulating the Rb-E2F1 pathway.

The present study demonstrates that GNL3L encodes a functional hydrophobic amino acid-rich nuclear export signal (NES) at the carboxyl-terminus. By using a heterokaryon assay, we demonstrate that GNL3L shuttles between nucleus and cytoplasm by a signal-mediated process. The export of GNL3L from the nucleus is sensitive to fungal metabolite, Leptomycin B (LMB) and is dependent on CRM1 mediated export pathway. Results from the protein-protein interaction experiments suggest that GNL3L interacts with export receptor, CRM1 through its C-terminal domain. Collectively, these data suggest that GNL3L encodes multiple functional nuclear import (NoLS and NLSs) as well as export signals (NES) (Fig 11A) for its subcellular distribution in different cellular compartments.

A variety of cargoes undergo CRM1-dependent transport, including nucleolar proteins, RNA and ribonucleo-protein subunits [36]. For example, Human NMD3, a nucleolar protein, binds to pre-60S ribosomal particles and mediates their export from the nucleus by CRM1-dependent pathway [13]. Recently, we have shown that GNL3L is critical for pre-nucleolar rRNA processing which is pre-requisite for ribosome assembly/biogenesis in the nucleolus. Interestingly, GNL3L complements the defects in growth and nuclear export of ribosomal subunits in Grn1-null *S*. *pombe* mutant [5]. These results suggest that the non-ribosomal proteins sequestered in the nucleolus may act as transport vehicles to deliver ribosomes to cytoplasm from the site of synthesis via nucleus for translation. One can speculate that the nuclear export of GNL3L could also play a role in ribosomal subunit export from nucleolus to cytoplasm in mammalian cells during cell proliferation. It is worth mentioning that although GNL3L interacts with CRM1, export of GNL3L wild type protein from HeLa nucleolus was not observed in the heterokaryon assay. This may be due to a) interaction of GNL3L with nucleolar proteins or other nucleolar structural components through its N-terminal NoLS, b) NoLS may be acting as a gating mechanism to regulate GNL3L cellular distribution, c) GNL3L might localize in different cellular compartments in a cell cycle dependent manner or d) slower export kinetics of the wild type GNL3L protein. Further experiments are warranted to define the molecular mechanism(s) of GNL3L export from the nucleolus.

The compartmentalization of cell contents employed by eukaryotes is an efficient mechanism to regulate cellular functions. The spatio-temporal requirement for a protein could be decided by the role it has to play at a particular phase of the cell cycle. On a similar note, a nucleolar non-ribosomal protein, GNL-1 is reported to be localized in different cellular compartments in cell cycle dependent manner [37]. In addition, The Yin-Yang 1 protein (YY1), a ubiquitously expressed zinc-finger transcription factor, accumulates in the nucleus during ‘S’ phase and nuclear export of YY1 is shown to be critical for the inhibition of cellular transformation and tumor growth [38]. Furthermore, phosphorylation of cyclin D1 by GSK-3β promotes its export by facilitating its association with CRM1 and the abrogation of phosphorylation results in its nuclear localization, thus promoting subcutaneous tumor formation in SCID mice [39]. In accordance with this, our data suggests that the inhibition of GNL3L export from the nucleus results in faster progression through ‘S’ phase, which is positively correlated with increased DNA synthesis (indicated by higher rate of BrdU incorporation). Collectively, our data suggest that GNL3L may regulate cell division in subcellular localization dependent manner.

Under normal physiological conditions, the G1-S transition (‘R’ or restriction point) is guarded by hypophosphorylated retinoblastoma protein which interacts with E2F1 and prevents its transcriptional activity. This leads to inhibition of transcription of E2F1 target genes, such as cyclin A and cyclin E, resulting in ‘S’ phase arrest (Fig 11B) Deregulation of this pathway potentially leads to uncontrolled cell proliferation and contributes to tumorigenesis. Hypophosphorylated Rb protein is an important E2F1 regulator which not only inhibits its activity but also prevents its degradation [40]. The phosphorylation of Rb is carried out by cyclin D-cdk4/6, cyclin E1-cdk2 and cyclin A-cdk2 complexes during ‘G1’ and ‘S’ phases of the cell cycles, respectively [41, 42]. The proposed model (Fig 11C) based on data from the present study suggests that the nuclear localization of GNL3L promotes cyclin D1-cdk4 association, which might lead to increased phosphorylation of Rb on serine 780. Hyperphosphorylation of Rb results in the release of E2F1 from the Rb/E2F1 inhibitory complex resulting in upregulation of cyclins A2 and E1 levels, which is critical for ‘S’ phase progression. In addition, our data suggest that GNL3L may directly influence E2F1 expression and regulate cell proliferation (Fig 9B and 9C; Fig 11C). Collectively, the present investigation provides evidence that nucleo-cytoplasmic shuttling of GNL3L could be one important regulatory mechanism for cell cycle control during cell proliferation.

## Supporting Information

S1 Fig Subcellular localization and GTP binding ability of wild type or G-domain mutants of GNL3L. (TIF)

S2 Fig Subcellular distribution of wild type and various deletion mutants of GNL3L. HeLa cells were transfected with wild type or N-terminal deletions of GNL3L and the subcellular localization was analyzed using confocal microscopy. Nucleolin was used as a nucleolar marker.
(TIF)

S3 Fig Quantitative distribution of wild type and variants of GNL3L in different cellular compartments. Subcellular distribution was quantified by analyzing the localization patterns of full length or indicated variants of GNL3L in one hundred GFP positive cells from three independent experiments. Error bar represents mean±S.D.
(TIF)

S1 Table List of primers used for the construction of GNL3L variants and real-time quantitative PCR analysis. (XLSX)
