# Supplementary figures and images for: GNL3L Is a Nucleo-Cytoplasmic Shuttling Protein: Role in Cell Cycle Regulation
Source: PLoS One. 2015 Aug 14;10(8):e0135845. doi: 10.1371/journal.pone.0135845 (PMC4537249; doi:10.1371/journal.pone.0135845)

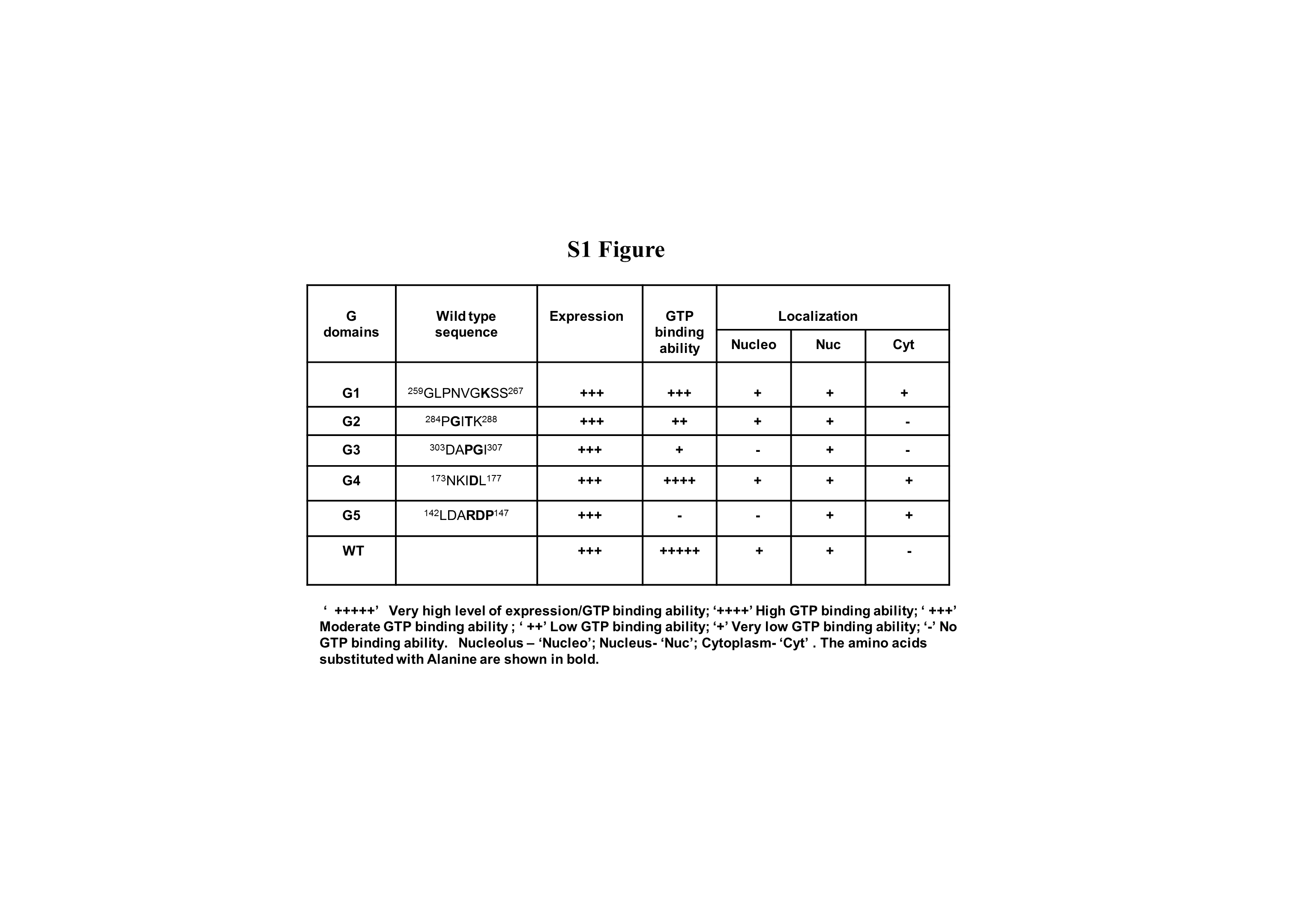

Supplement: S1 Fig — (TIF) [file pone.0135845.s001.tif]

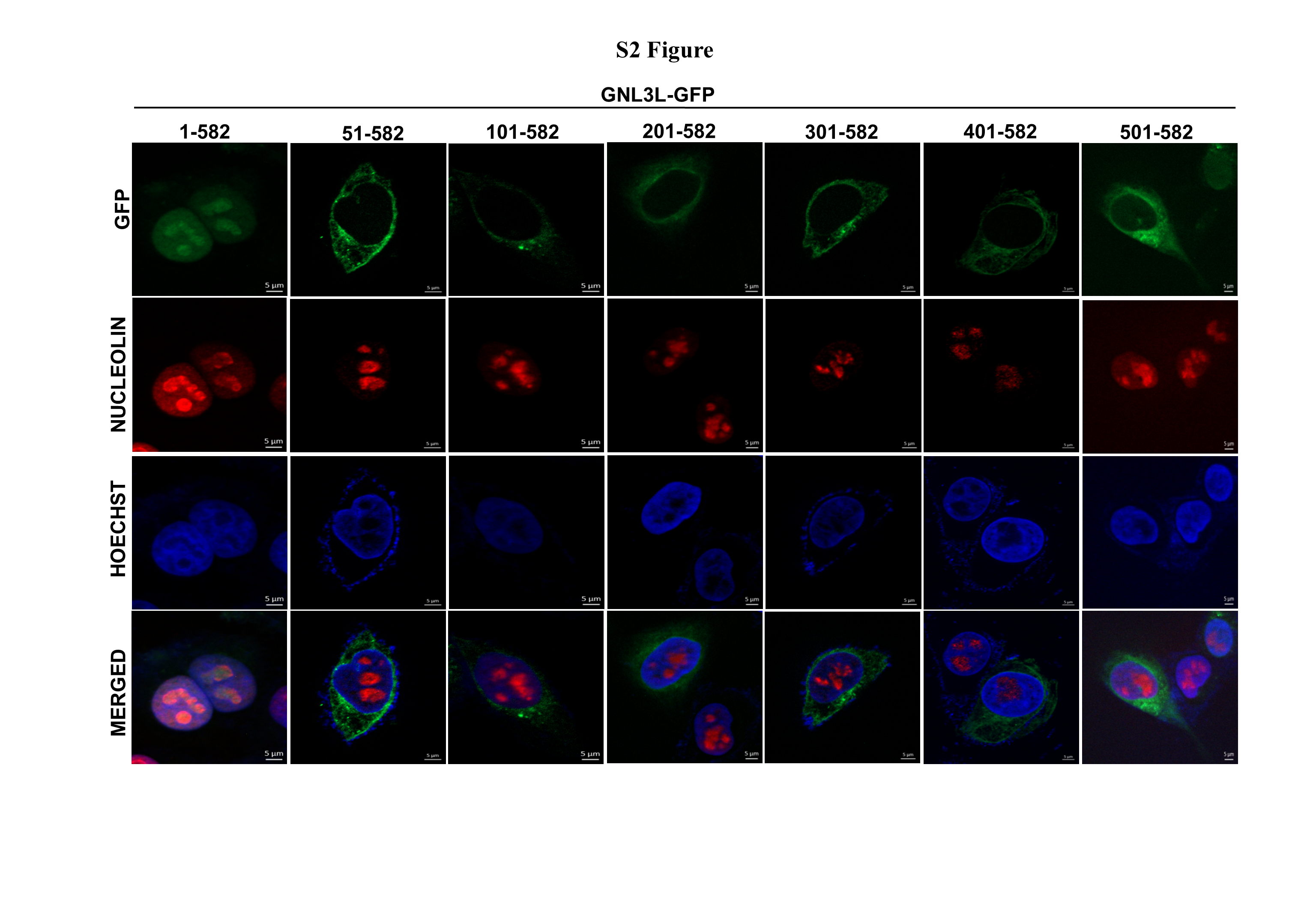

Supplement: S2 Fig — HeLa cells were transfected with wild type or N-terminal deletions of GNL3L and the subcellular localization was analyzed using confocal microscopy. Nucleolin was used as a nucleolar marker. (TIF) [file pone.0135845.s002.tif]

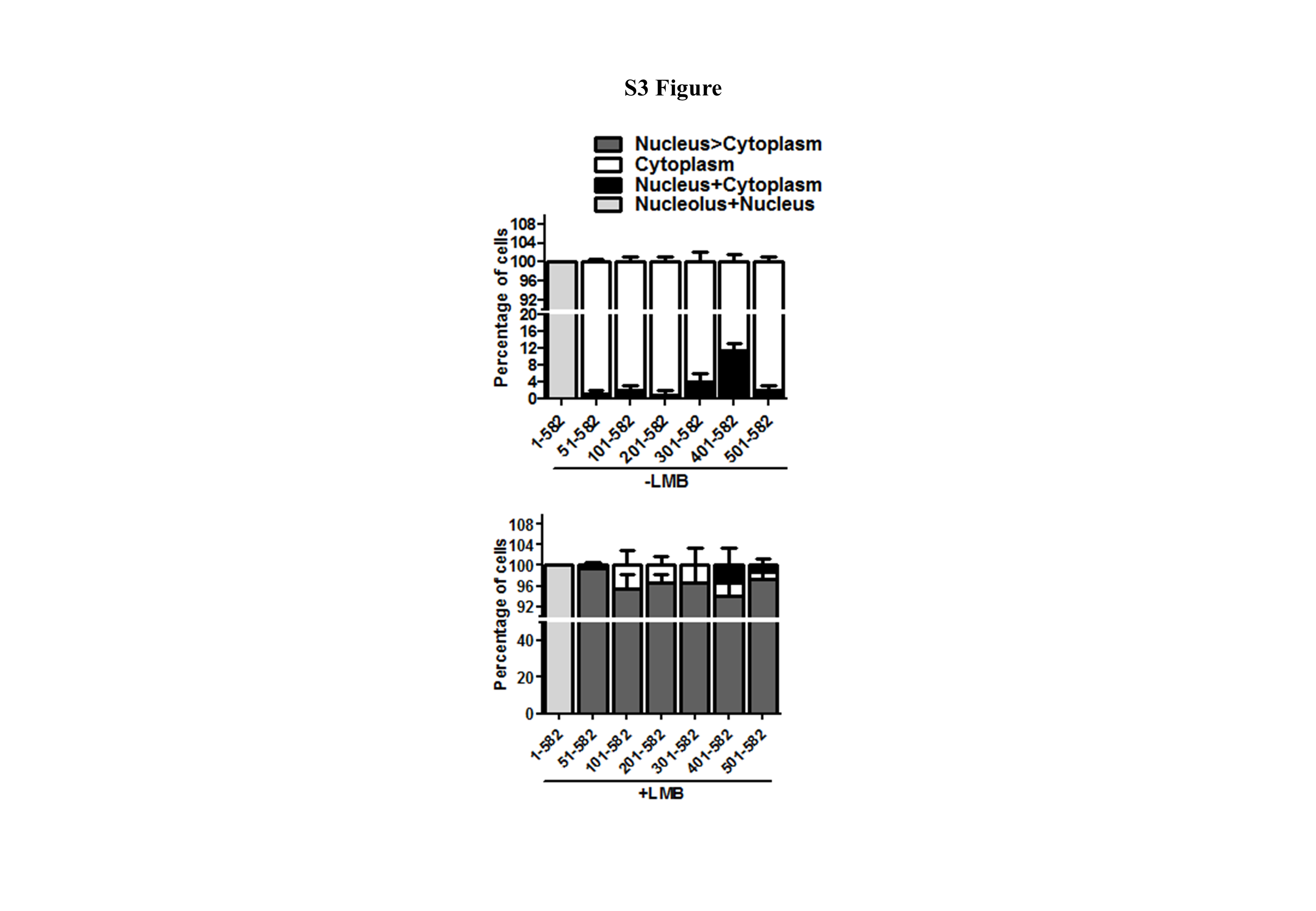

Supplement: S3 Fig — Subcellular distribution was quantified by analyzing the localization patterns of full length or indicated variants of GNL3L in one hundred GFP positive cells from three independent experiments. Error bar represents mean±S.D. (TIF) [file pone.0135845.s003.tif]
